# High revision rates and mortality after distal femoral replacement for periprosthetic distal femoral fractures: analysis from the German Arthroplasty Registry (EPRD)

**DOI:** 10.1007/s00590-023-03582-2

**Published:** 2023-07-27

**Authors:** Jörg Lützner, Oliver Melsheimer, Arnd Steinbrück, Anne Elisabeth Postler

**Affiliations:** 1 University Center of Orthopaedics, Trauma and Plastic Surgery, University Hospital Carl Gustav Carus, TU Dresden, Dresden, Germany; 2 German Arthroplasty Registry (EPRD), Berlin, Germany; 3 Center for Orthopaedic Surgery, Augsburg, Germany

**Keywords:** Periprosthetic distal femur fracture, TKA, TKR, Distal femoral replacement, Mortality, Revision

## Abstract

**Purpose:**

This study was initiated to analyze the outcome after distal femoral replacement (DFR) for periprosthetic distal femoral fractures (PDFF).

**Methods:**

Data from the German Arthroplasty Registry (EPRD) were analyzed. A total of 626 patients could be identified with a DFR for PDFF. Mean age was 78.8 years, and 84.2% were female. Revisions and mortality were analyzed and compared with patient groups with a similar procedure (revision total knee arthroplasty) or similar general condition (fracture total hip arthroplasty, hip hemiarthroplasty). Matched-pair-analyses were performed.

**Results:**

Within one year after surgery, 13.2% of the patients had died and further 9.4% were revised. Within four years, 32.7% had died and 19.7% were revised. Revisions were nearly twice as high as in the comparison groups. Periprosthetic infection (PJI) was the most frequent cause for revision, resulting in a PJI rate of 12.8%, which was lower in the comparison groups. Mortality after DFR was as similar high as after fracture hip arthroplasty.

**Conclusion:**

PDFF are a serious injury, and the necessary surgical treatment has a high risk of complications. Every third patient after DFR for PDFF had died and every fifth patient needed revision within 4 years after surgery. Efforts should be undertaken to provide optimal treatment to these high-risk patients to reduce unfavorable outcomes.

**Level of evidence:**

III.

**Registration of clinical trials:**

As this is a registry-derived study of data of the German Arthroplasty Registry (EPRD), no registration was performed.

## Introduction

**Fig. 1:**
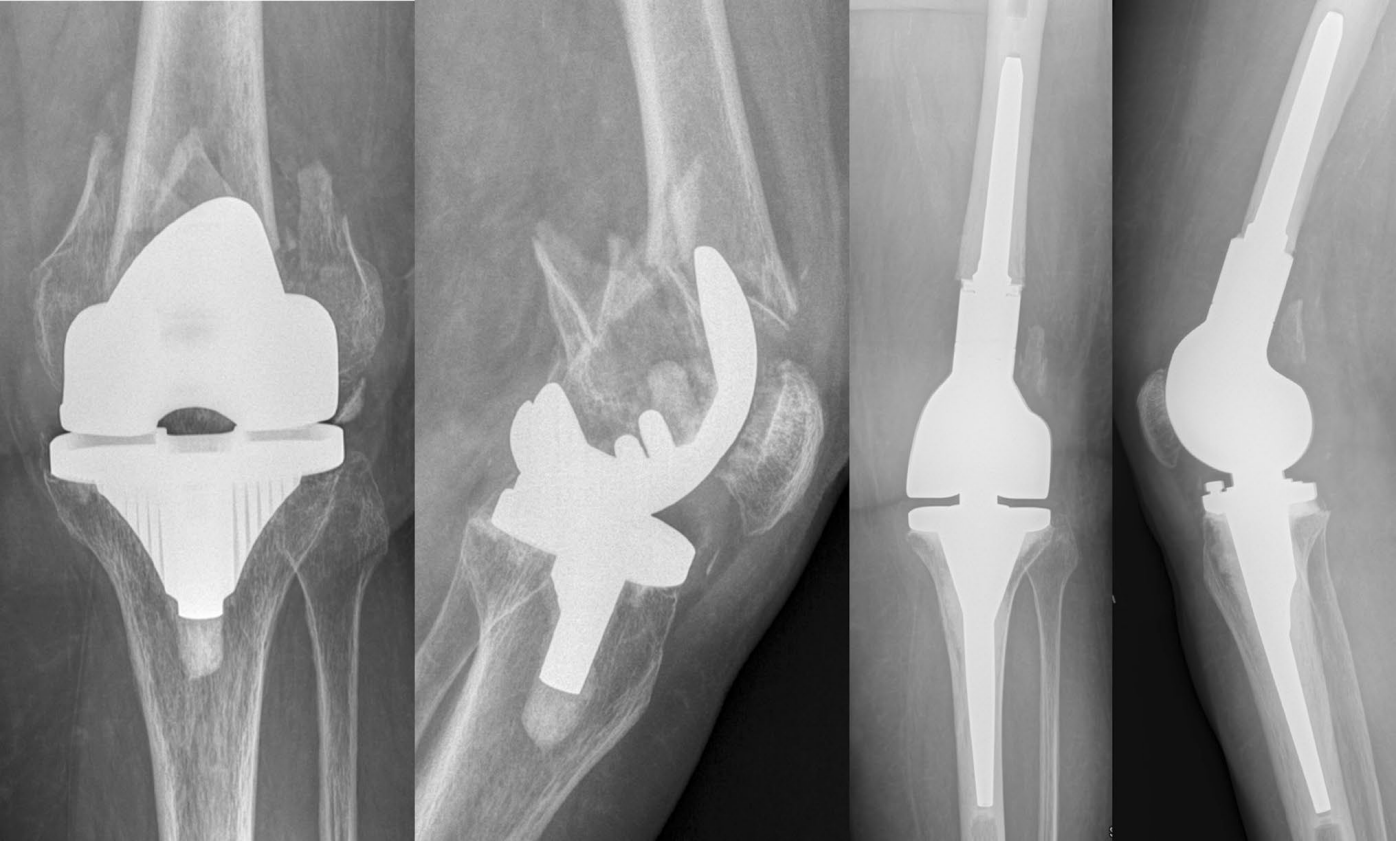
78-year-old female patient with a PDFF and revision to DFR, immediate weight-bearing on crutches after surgery, after 3 years still walking with one cane, no signs of loosening

As a result of aging populations and increasing numbers of knee arthroplasties worldwide, the numbers of periprosthetic fractures are rising accordingly. Among them, periprosthetic distal femoral fractures (PDFF) are frequent. The incidence has been estimated at 2.4 per 100,000 per year [1]. Periprosthetic femoral fractures are challenging injuries as mostly geriatric patients with serious comorbidities are affected. These patients are at high risk for complications due to their general condition [2–5]. Furthermore, bone quality is often reduced, making the treatment even more difficult. Depending on the fracture type, bone quality and fixation of the total knee arthroplasty (TKA), there are two different treatment options: fixation or revision arthroplasty [6]. For well-fixed TKA and acceptable bone-stock, fixation is usually performed. In cases with comminution/poor bone-stock and/or loose TKA, revision arthroplasty is necessary [7, 8]. In these cases, distal femoral replacement (DFR) can be used. This is a major but straightforward surgery, usually allowing the patient immediate full weight-bearing (Fig. 1). In geriatric patients, full weight-bearing is of crucial importance, as many of them may not be able to comply with partial weight-bearing and immobility is a serious risk for complications [9–11]. Sometimes both treatment options are possible and the surgeon needs to decide which option is best for the individual patient. To enable evidence-based shared decision making both, the surgeon and the patient need to know which risks are associated with the surgery and what outcome can be expected.

To date, four meta-analyses have been published on this topic [12–15]. These meta-analyses included only small case series with level IV evidence, making it difficult to estimate the real-world revision rate and mortality. All four meta-analyses acknowledged the limited data, a high risk of bias and recommended further studies. Recently, two analyses from the Australian Joint Replacement Registry (AOANJRR) reported about DFR in PDFF [16] and native knee fractures [17]. Despite being one of the oldest Arthroplasty registry, the number of cases for analysis was limited.

The German Arthroplasty Registry (EPRD) receives reliable information on revision rates and mortality from hospitals and health insurance companies once the patient is entered into the registry database [18]. These data can therefore be used to increase the knowledge about these serious injuries.

The primary aim of this study was to investigate the revision rate and mortality after DFR for PDFF in a large cohort from the EPRD. Secondary, a comparison of the results with EPRD data of patient groups with a similar surgical procedure (revision total knee arthroplasty, RTKA) or similar general condition (fracture total hip arthroplasty, FTHA, hip hemiarthroplasty, HA) was performed.

## Materials and methods

The EPRD started data acquisition in November 2012 and has currently a total number of more than 2 million hip and knee replacements in its database. It includes primary and revision arthroplasty surgeries. Although participation is voluntary, about 70% of all hip and knee arthroplasties in Germany are covered [19]. Prior to enrollment in the registry, all patients provided informed consent at the participating hospital. Once entered into the registry database, the follow-up of an arthroplasty is nearly complete because data on revisions are obtained not only from hospitals, but additionally from health insurance companies. Data about comorbidities and death are obtained from health insurance companies on a regular basis [18].

Patients were included in this study if they were entered with (1) TKA revision and (2) revision cause of periprosthetic fracture and (3) distal femoral replacement was implanted during that revision. From a total number of 37,591 TKA revisions which were registered in the EPRD until 2021, 626 were identified with a diagnosis of periprosthetic fracture in which a DFR was used as the initial procedure for that fracture. Patients with a DFR but different diagnosis (e.g., pseudarthrosis after failed fracture fixation) were not included. For comparison to a similar surgical procedure, RTKA for aseptic loosening was used (*n* = 8,761). For comparison to patient groups with a similar general condition, total hip arthroplasty for femoral neck fractures (FTHA, *n* = 23,578) and hip hemiarthroplasty (HA, *n* = 47,901) were used.

Events of interest were revisions and mortality. Revisions were further discriminated into periprosthetic joint infection and aseptic revisions.

There were significant differences between the groups regarding age, gender, BMI (body mass index) and comorbidities (weighted Elixhauser score) which are described in Table 1. To reduce the bias by these factors a Mahalanobis-Distance-Matching was performed. After matching there were still significant differences between groups regarding age, but these differences were small and were therefore considered as clinically not relevant.

**Table 1:** Characteristics of patient groups, crude data and after Mahalanobis-Distance-Matching (1:1 RTKA, 1:6 FTHA and 1:6 HA)

Crude data (mean (SD) or relative frequencies)	DFR *n* = 626	RTKA *n* = 8761	FTHA *n* = 23,578	HA *n* = 47,901	*p *value
Age at surgery (years)	78.8 (9.4)	70.3 (9.9)	74.9 (9.9)	83.7 (7.7)	< 0.001
Female gender (%)	84.2	65.9	70.3	71.7	< 0.001
BMI (kg/m^2^)	29.0 (5.8)	31.2 (6.1)	25.2 (4.4)	24.7 (4.2)	< 0.001
Elixhauser score	6.1 (7.3)	1.8 (5.3)	5.4 (6.9)	8.7 (7.9)	< 0.001

After matching	DFR *n* = 626	RTKA *n* = 626	FTHA *n* = 3756	HA *n* = 3756	*p* value
Age at surgery (years)	78.8 (9.4)	78.2 (8.8)	78.6 (9.1)	79.2 (9.0)	0.018
Female gender (%)	84.2	84.2	84.2	84.2	1.000
BMI (kg/m^2^)	29.0 (5.8)	29.1 (5.7)	28.8 (5.6)	28.7 (5.5)	0.109
Elixhauser score	6.1 (7.3)	5.9 (7.1)	6.1 (7.2)	6.4 (7.0)	0.419

*BMI* body mass index, *DFR* distal femoral replacement, *FTHA* fracture total hip arthroplasty, *HA* hip hemiarthroplasty, *RTKA* revision total knee arthroplasty, *SD* standard deviation

### Statistical analysis

Data description was based on absolute and relative frequencies and means (standard deviation, SD) or 95% confidence interval (CI)). Patients were followed-up with respect to revision, death or amputation including the replaced joint. Kaplan–Meier survival analysis was performed to estimate cumulative incidence for revision for any reason (cumulative revision rate, CRR) and mortality. Patients with incomplete follow-up, those who did not require a revision up to the end of the follow-up period or prior to their death or amputation, have been regarded as being ‘censored’ at those times. Differences between groups were tested using the log-rank test. Significance level was set to 0.05. All analyses were performed using R statistical software (R Foundation for Statistical Computing, Vienna, Austria).

## Results

A total of 626 DFR for PDFF were analyzed. These surgeries were performed in 254 hospitals. In the comparison groups, the respective surgeries were performed in a higher number of different hospitals (RTKA 614 hospitals, FTHA 632 hospitals, HA 556 hospitals). In most cases, DFR was cemented (66.5%), but in 25.7% cementless stems were used for femur and tibia fixation. Demographic data of the DFR group and comparison groups are presented in Table 1.

**Fig. 2:**
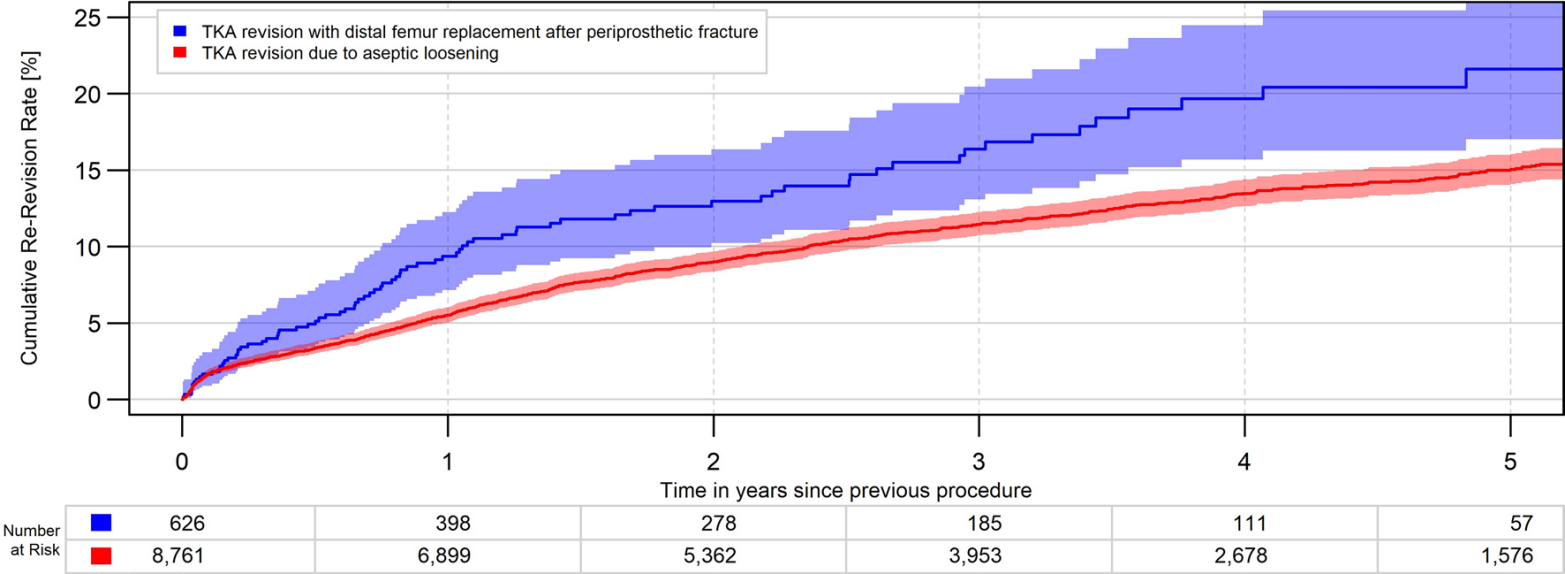
All-cause revision rate for DFR for PDFF and RTKA for aseptic loosening (*p* = 0.006)

**Fig. 3:**
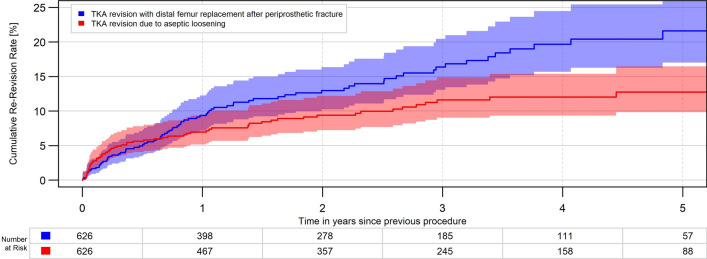
All-cause revision rate for DFR for PDFF and RTKA for aseptic loosening after 1:1 matching (*p* = 0.02)

The analysis demonstrated a high all-cause cumulative revision rate (CRR) of 9.4% for the DFR group within the first year, which was lower in the unmatched comparison groups: RTKA (5.5%), FTHA (6.1%) and HA (4.6%). There was no relevant change in revision rates after matching for age, gender, BMI and comorbidities (Table 2, Figs. 2 and 3).

**Table 2:** cumulative revision rate in percent, crude data and after matching 1:1 to RTKA and 1:6 to FTHA and HA

Crude data (mean (95% CI)	DFR *n* = 626	RTKA *n* = 8761	FTHA *n* = 23,578	HA *n* = 47,901
1 year	9.4 (7.2, 12.2)	5.5 (5.0, 6.0)	6.1 (5.8, 6.4)	4.6 (4.4, 4.8)
2 years	13.0 (10.2, 16.4)	9.0 (8.4, 9.7)	6.7 (6.4, 7.0)	4.9 (4.7, 5.1)
3 years	16.4 (13.1, 20.4)	11.5 (10.7, 12.2)	7.1 (6.8, 7.5)	5.1 (4.8, 5.3)
4 years	19.7 (15.7, 24.5)	13.5 (12.6, 14.4)	7.5 (7.1, 7.9)	5.2 (5.0, 5.5)

After matching	*n* = 626	*n* = 626	*n* = 3756	*n* = 3756
1 year	9.4 (7.2, 12.2)	7.0 (5.2, 9.3)	6.8 (6.0, 7.7)	4.8 (4.1, 5.5)
2 years	13.0 (10.2, 16.4)	9.4 (7.2, 12.2)	7.3 (6.5, 8.3)	5.2 (4.4, 6.0)
3 years	16.4 (13.1, 20.4)	11.6 (9.0, 14.9)	7.6 ( 6.7, 8.6)	5.3 (4.5, 6.1)
4 years	19.7 (15.7, 24.5)	12.0 (9.4, 15.4)	8.1 (7.2, 9.2)	5.6 (4.8, 6.5)

*CI* confidence interval, *DFR* distal femoral replacement, *FTHA* fracture total hip arthroplasty, *HA* hip hemiarthroplasty, *RTKA* revision total knee arthroplasty

**Fig. 4:**
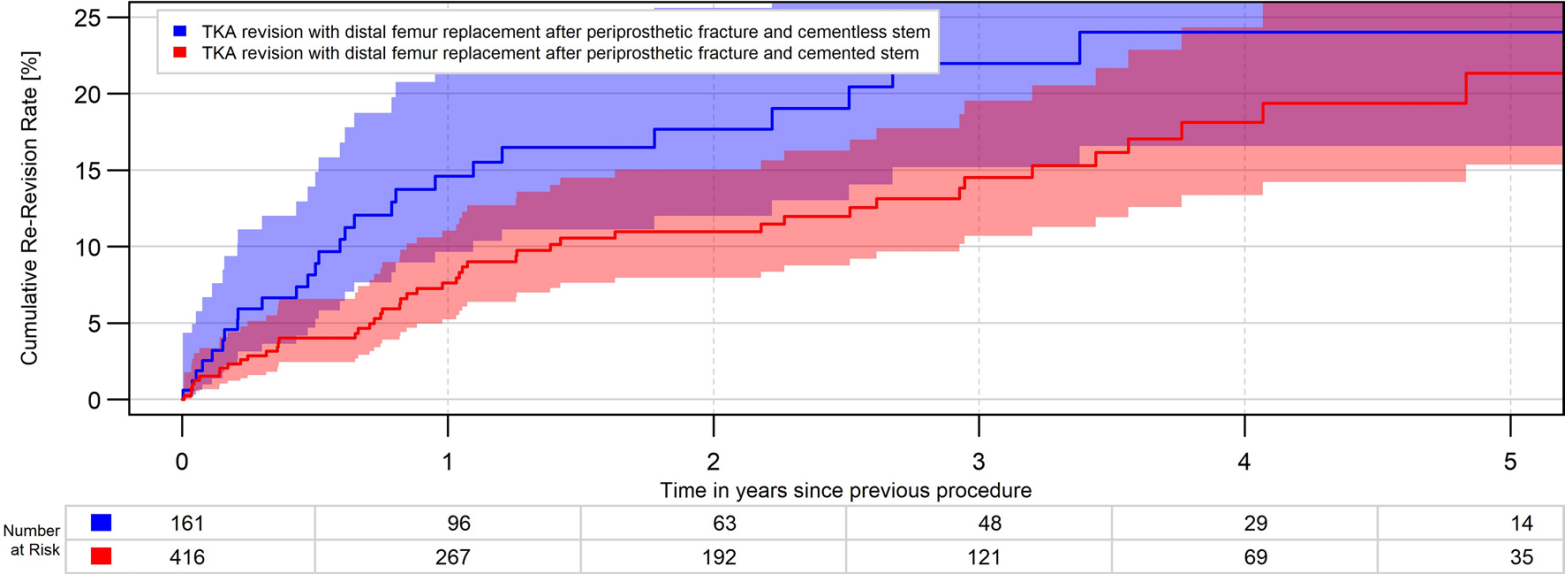
All-cause revision rate for cemented and cementless DFR fixation (*p* = 0.07)

In the DFR group, revision rates were not statistically significant different between cemented and cementless stem fixation (*p* = 0.07, Fig. 4).

These revision rates were mainly caused by periprosthetic joint infection (PJI), which accounted for 62.5% of all revisions in the DFR group, resulting in a PJI rate of 12.8%. PJI was also the leading revision cause in the comparison groups: 41.3% in RTKA, 34.8% in FTHA and 48.7% in HA.

**Fig. 5:**
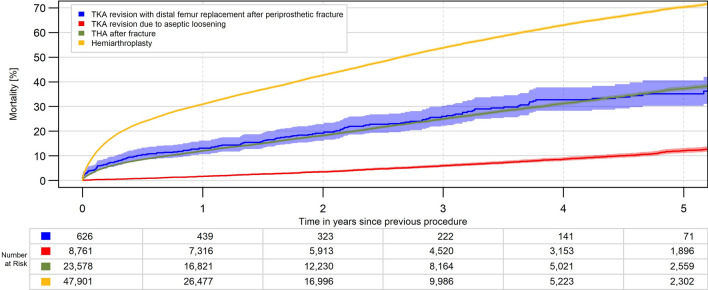
Mortality after DFR for PDFF, RTKA for aseptic loosening, FTHA and HA (*p* < 0.001)

**Fig. 6:**
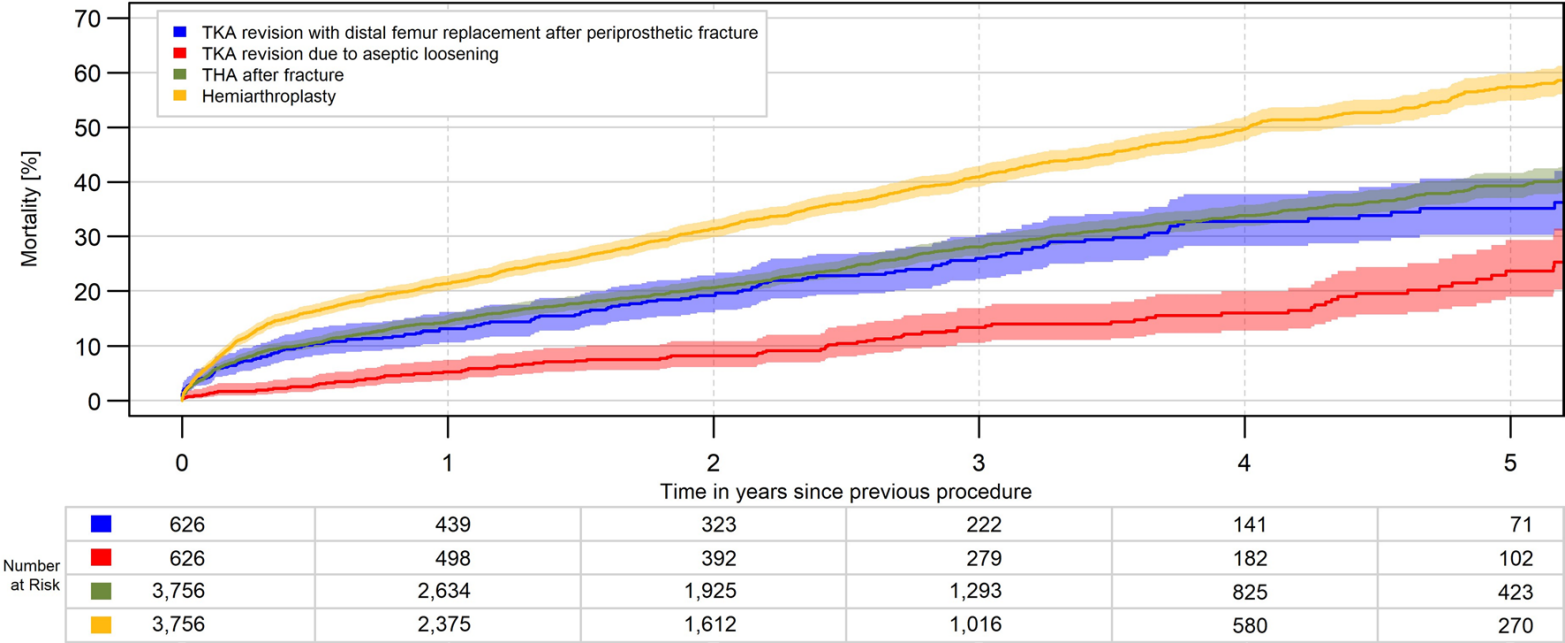
Mortality after DFR for PDFF, RTKA for aseptic loosening, FTHA and HA after matching 1:1 to RTKA and 1:6 to FTHA and HA (*p* < 0.001)

Cumulative mortality was high after DFR (13.2% after 1 year, 32.7% after 4 years). This was considerably lower in RTKA (1.7% after one, 8.6% after 4 years), equivalent in FTHA (12.0% after one, 31.2% after 4 years) but much higher in HA (30.9% after one, 63.0% after 4 years). After matching to quite similar groups regarding age, gender, BMI and comorbidities, the differences between groups were lower but remained significant (Table 3, Figs. 5 and 6).

**Table 3:** cumulative mortality in percent, crude data and after matching 1:1 to RTKA and 1:6 to FTHA and HA

Crude data (mean (95% CI)	DFR *n* = 626	RTKA *n* = 8761	FTHA *n* = 23,578	HA *n* = 47,901
1 year	13.2 (10.7, 16.2)	1.7 (1.4, 2.0)	12.0 (11.6, 12.4)	30.9 (30.5, 31.3)
2 years	19.2 (16.1, 22.8)	3.5 (3.1, 4.0)	18.2 (17.7, 18.8)	42.7 (42.3, 43.3)
3 years	26.0 (22.2, 30.3)	6.0 (5.4, 6.6)	25.0 (24.3, 25.6)	53.8 (53.2, 54.3)
4 years	32.7 (28.3, 37.7)	8.6 (7.9, 9.4)	31.2 (30.5, 32.0)	63.0 (62.4, 63.6)

After matching	*n* = 626	*n* = 626	*n* = 3756	*n* = 3756
1 year	13.2 (10.7, 16.2)	5.3 (3.8, 7.5)	14.4 (13.3, 15.6)	21.4 (20.1, 22.8)
2 years	19.2 (16.1, 22.8)	8.2 (6.2, 10.9)	20.6 (19.3, 22.1)	31.5 (29.9, 33.1)
3 years	26.0 (22.2, 30.3)	13.4 (10.6, 16.9)	28.1 (26.4, 29.8)	40.9 (39.1, 42.8)
4 years	32.7 (28.3, 37.7)	16.0 (12.7, 20.0)	33.8 (31.9, 35.8)	49.7 (47.6, 51.9)

*CI* confidence interval, *DFR* distal femoral replacement, *FTHA* fracture total hip arthroplasty, *HA* hip hemiarthroplasty, *RTKA* revision total knee arthroplasty

## Discussion

PDFF are serious injuries which usually need surgical treatment. The goal is to save as much bone as possible and to enable weight-bearing. If the fracture and the bone-stock allow, fixation is usually preferred. If this is not possible revision arthroplasty, often with a DFR is necessary. This large cohort study from the EPRD investigated the most serious complications of DRF for PDFF, revision and mortality.

The revision rate in this study was nearly 20% after 4 years. This is slightly higher than in previous meta-analysis. Wadhwa et al. [15] found a reoperation/revision rate of 14.2% after mean 35 month of follow-up. Quinci et al. [13] reported a reoperation rate of 19%. In the studies included by Rubinger et al. [14] reoperation rates ranged from 0 to 45%. Recent analysis from the AOANJRR reported revision rates of 12% for DFR in PDFF after 6 years [16] and 12% after 4 years in native knee fractures [17]. As in the AOANJRR, this study showed no significant difference between cemented and cementless stem fixation which might be caused by the low number of cementless DFR. Taking into consideration the predominantly female geriatric population with often limited bone quality, it seems reasonable to consider cemented fixation as the standard.

The main cause of revision in this study in the DFR group was PJI with 62.5% of all revisions, resulting in a PJI rate of 12.8%. Further discrimination of the aseptic revision causes was not possible because some revisions were reported by health insurance companies only. In these cases, only infection and aseptic revision could be distinguished. This PJI rate is higher than previously reported. Mechas et al. [12] reported a meta-effect estimate of 4.3% (95% CI 2.2–8.2), Wadhwa et al. [15] of 5.7% and Quinci et al. [13] of 9%. In all three meta-analyses, the limited evidence due to the low quality and case numbers of the included studies, publication bias and loss to follow-up was acknowledged. It was suggested to consider these rates as the lower bound estimate. This explains the higher rate in this EPRD-derived study, which ensured nearly complete follow-up of arthroplasties. However, in the registry studies from the AOANJRR about DFR the infection rate was 4.4% for PDFF (accounting for 37% of all revisions) and only 2.6% for native knee fractures (accounting for 25% of all revisions) [16, 17]. This large difference between Australia and Germany may be explained by the number of hospitals performing that surgeries. While DFR was performed in 254 hospitals in Germany—which results in mean in only 2.5 cases per hospital—this might be different if a larger number of surgeries are performed in specialized hospitals in Australia. The patients from the AOANJRR were slightly younger, but otherwise similar with regard to gender and BMI. Comorbidities could not be compared directly between the AOANJRR and EPRD cohorts because ASA score has been commenced in the EPRD not until 2020 and was therefore not available for most patients. However, mortality was considerably higher in the EPRD cohort which might indicate more comorbidities and frailty. These are also well-known risk factors for PJI.

Mortality was unsurprisingly high in this geriatric population with 13.2% after one year. Much higher mortality rates up to 36% in the first year have been reported in case series [20]. This is comparable to the meta-analysis of Mechas et al. [12] who reported a pooled 1-year mortality of 10% (95% CI 6–18). Quinci et al. [13] reported a 1-year mortality of 6% and within mean 30-month follow-up of 13%. These mortality rates are higher than for many oncologic diseases and efforts should be undertaken to improve recovery and survival of the patients. Crucial points for improvement could be the recognition of the severity of these injuries as well as timing of surgery and immediate postoperative mobilization with unrestricted weight bearing [21]. Mortality was much less in the Australian registry study with patient survival of 97% at 5 years and 83% at 10 years, respectively. As mentioned above, these differences might be caused by differences in the patient populations regarding age and comorbidities.

To account for differences in patient populations, DFR for PDFF was compared to a similar but elective procedure (RTKA) and to similar population groups with a non-elective surgery (FTHA, HA). After matching to similar patient groups regarding age, gender, BMI and comorbidities, revisions at any time were nearly twice as high in the DFR group compared to elective RTKA. This demonstrates the importance of careful patient preparation for a major surgery and might be a point for improvement in PDFF. Mortality was two to three times higher in DFR compared to elective RTKA and equivalent to FTHA in femoral neck fractures as a typical geriatric trauma. The similar general health condition of patients who sustain a hip fracture or a periprosthetic femur fracture has recently described in a British cohort [5]. Only HA had an even higher mortality. This can be explained by the fact that usually only frail patients with a limited life expectancy receive that type of hip arthroplasty.

The strength of the presented study is its sample size, which is higher than published meta-analysis and registry studies so far. Furthermore, the nearly complete follow-up allows for reliable and realistic data. We acknowledge some limitations. In general, registry-based studies lack detailed information about the included patients. It is therefore unknown how decision for the revision to DFR was made and if alternative treatment options would have been available. The EPRD is a voluntary arthroplasty registry. Therefore, not all DFR performed in Germany was included. However, data can be considered representative as about 70% of all arthroplasties were covered and hospitals which do not provide data are smaller hospitals which usually do not perform revision surgery. Only information on revisions and mortality could be analyzed. Further outcomes (e.g., function, ambulation) are not entered into the registry but are of interest for shared-decision making. As only arthroplasties are recorded in the EPRD we were not able to compare DFR to fracture fixation. We were not able to further discriminate aseptic revision causes which is of interest to improve the results. Therefore, further studies from centers with relevant numbers of PDFF and complete follow-up data with more detailed information are needed.

In conclusion, periprosthetic distal femoral fractures are serious injuries and the necessary surgical treatment is associated with high risks. This registry-based study demonstrated a high mortality which was similar to hip fracture arthroplasty. Revisions were nearly twice as high as in comparable elective revision TKA. Efforts should be undertaken to provide optimal treatment to these high-risk patients to reduce the revision rate and mortality.

## References

[1] Elsoe R, Ceccotti AA, Larsen P (2018). Population-based epidemiology and incidence of distal femur fractures. Int Orthop.

[2] Moloney GB, Pan T, Van Eck CF, Patel D, Tarkin I (2016). Geriatric distal femur fracture: are we underestimating the rate of local and systemic complications?. Injury.

[3] Streubel PN (2013). Mortality after periprosthetic femur fractures. J Knee Surg.

[4] Bhattacharyya T, Chang D, Meigs JB, Estok DM, Malchau H (2007). Mortality after periprosthetic fracture of the femur. J Bone Joint Surg Am.

[5] Compose Study Team (2022). Epidemiology and characteristics of femoral periprosthetic fractures: data from the characteristics, outcomes and management of periprosthetic fracture service evaluation (COMPOSE) cohort study. Bone Joint J.

[6] Quinzi DA, Childs S, Lipof JS, Soin SP, Ricciardi BF (2020). The treatment of periprosthetic distal femoral fractures after total knee replacement: a critical analysis review. JBJS Rev.

[7] Girgis E, McAllen C, Keenan J (2018). Revision knee arthroplasty using a distal femoral replacement prosthesis for periprosthetic fractures in elderly patients. Eur J Orthop Surg Traumatol.

[8] Laubach LK, Sharma V, Krumme JW, Larkin K, Satpathy J (2023). Novel classification system for periprosthetic distal femoral fractures: a consideration for comminution. Eur J Orthop Surg Traumatol.

[9] Ottesen TD, McLynn RP, Galivanche AR, Bagi PS, Zogg CK, Rubin LE (2018). Increased complications in geriatric patients with a fracture of the hip whose postoperative weight-bearing is restricted an analysis of 4918 patients. Bone Joint J.

[10] Keenan OJF, Ross LA, Magill M, Moran M, Scott CEH (2021). Immediate weight-bearing is safe following lateral locked plate fixation of periprosthetic distal femoral fractures. Knee Surg Relat Res.

[11] Langenhan R, Trobisch P, Ricart P, Probst A (2012). Aggressive surgical treatment of periprosthetic femur fractures can reduce mortality: comparison of open reduction and internal fixation versus a modular prosthesis nail. J Orthop Trauma.

[12] Mechas CA, Isla AE, Abbenhaus EJ, Landy DC, Duncan ST, Selby JB (2022). Clinical outcomes following distal femur replacement for periprosthetic distal femur fractures: a systematic review and meta-analysis. J Arthroplast.

[13] Quinzi DA, Ramirez G, Kaplan NB, Myers TG, Thirukumaran CP, Ricciardi BF (2021). Early complications and reoperation rates are similar amongst open reduction internal fixation, intramedullary nail, and distal femoral replacement for periprosthetic distal femur fractures: a systematic review and meta-analysis. Arch Orthop Trauma Surg.

[14] Rubinger L, Khalik HA, Gazendam A, Wolfstadt J, Khoshbin A, Tushinski D (2021). Very distal femoral periprosthetic fractures: replacement versus fixation: a systematic review. J Orthop Trauma.

[15] Wadhwa H, Salazar BP, Goodnough LH, Van Rysselberghe NL, DeBaun MR, Wong HN (2022). Distal femur replacement versus open reduction and internal fixation for treatment of periprosthetic distal femur fractures: a systematic review and meta-analysis. J Orthop Trauma.

[16] Aebischer AS, Hau R, de Steiger RN, Holder C, Wall CJ (2022). Distal Femoral replacement for periprosthetic fractures after TKA: Australian orthopedic association national joint replacement registry review. J Arthroplast.

[17] Aebischer AS, Hau R, de Steiger RN, Holder C, Wall CJ (2022). Distal femoral arthroplasty for native knee fractures: results from the Australian orthopaedic association national joint replacement registry. Bone Joint J.

[18] Jansson V, Grimberg A, Melsheimer O, Perka C, Steinbrück A (2019). Orthopaedic registries: the German experience. EFORT Open Rev.

[19] Endoprothesenregister Deutschland (EPRD) (2022) Jahresbericht 2021. https://www.eprd.de/fileadmin/user_upload/Dateien/Publikationen/Berichte/Jahresbericht2021_2021-10-25_F.pdf. Accessed 26 July 2022.

[20] Pujol O, Joshi-Jubert N, Nunez JH, Pijoan J, Castellet E, Minguell J (2023). High reoperation and mortality rate after distal femoral replacement for periprosthetic knee fracture in the elderly. Eur J Orthop Surg Traumatol.

[21] Bliemel C, Rascher K, Knauf T, Hack J, Eschbach DA, Aigner R (2021). Early surgery does not improve outcomes for patients with periprosthetic femoral fractures—results from the registry for geriatric trauma of the German trauma society. Medicina.

